# *Psychrobacter arenosus* Bacteremia after Blood Transfusion, France

**DOI:** 10.3201/eid1907.121599

**Published:** 2013-07

**Authors:** Yvan Caspar, Christine Recule, Patricia Pouzol, Bruno Lafeuillade, Marie-Reine Mallaret, Max Maurin, Jacques Croize

**Affiliations:** Centre Hospitalier Universitaire de Grenoble, Grenoble, France (Y. Caspar, C. Recule, P. Pouzol, M.-R. Mallaret, M. Maurin, J. Croize);; Université Joseph Fourier-Grenoble 1, Grenoble, France (Y. Caspar, C. Recule, P. Pouzol, M.-R. Mallaret, M. Maurin, J. Croize);; Etablissement Français du Sang, La Tronche, France (B. Lafeuillade)

**Keywords:** Psychrobacter arenosus, bacteria, bacteremia, transfusion-transmitted infection, blood transfusion, France

## Abstract

We report a case of transfusion-associated bacteremia caused by *Psychrobacter arenosus*. This psychrotolerant bacterium was previously isolated in 2004 from coastal sea ice and sediments in the Sea of Japan, but not from humans. *P. arenosus* should be considered a psychrotolerant bacterial species that can cause transfusion-transmitted bacterial infections.

Bacteria are the leading cause of transfusion-transmitted infections (*1*). Contamination occurs more frequently in platelet concentrates than in erythrocyte units, especially because of different storage conditions (20°C–24°C for platelet concentrates vs. 1°C–6°C for erythrocyte units). However, several bacterial species are able to grow at 4°C (*1**–**3*). We report a case of transfusion-transmitted bacterial infection caused by *Psychrobacter arenosus*, an environmental psychrotolerant and halotolerant bacterium.

## The Patient

In October 2009, a 58-year-old man was admitted to Grenoble University Hospital (Grenoble, France) for a blood transfusion because of severe anemia. Idiopathic medullary aplasia had been diagnosed in 1997, and he had had grade 3 myelofibrosis since 2006. He had been receiving palliative care since November 2007, and received transfusions of erythrocyte units every 3 weeks. On October 27, 2009, he received 3 erythrocyte units (at 8:30 am, 10:30 am, and 12:15 pm). While receiving the third unit, he became febrile (temperature of 38°C that rapidly increased to 40°C) and had chills and headache. The transfusion was stopped and the patient transferred to the Department of Internal Medicine. At examination, there was no hypotension, jaundice, or red urine.

Standard laboratory testing showed no ABO incompatibility, hemoglobinemia, hemoglobinuria, and coagulation disorders. According to recommendations of the Agence Nationale de Sécurité du Médicament (Saint-Denis, France), 3 sets of aerobic and anaerobic blood cultures (Bactec; Becton Dickinson, Pont de Clay, France) for the recipient (1 immediately and 2 others 4 hours later) and the remaining part of the third erythrocyte unit were sent to the bacteriology laboratory for culture. Gram staining of a blood smear prepared from the third erythrocyte unit showed a large number (≈10^6^ CFU/mL) of gram-variable coccobacilli.

Samples were placed on Columbia blood agar (bioMérieux, Marcy L’Etoile, France) and incubated at 37°C in anaerobic or 5% CO_2_–enriched atmospheres. Sample inoculated into blood culture bottles were incubated at 37°C under aerobic and anaerobic conditions (Figure). The aerobic blood culture bottle of the first sample obtained from the recipient and aerobic cultures of the third erythrocyte unit enabled isolation of the same gram-variable coccobacilli after incubation for 48 hours (Figure). Colonies obtained on Columbia blood agar were monomorphic, small, and gray, and had positive results for oxidase and catalase tests. Phenotypic traits of the bacterial strains isolated from the blood of the patient and the erythrocyte unit were similar, but identification using the Vitek2 Gram negative card and API 20E, API 20NE, and ID 32 GN Kits (bioMérieux) was not successful.

**Figure:**
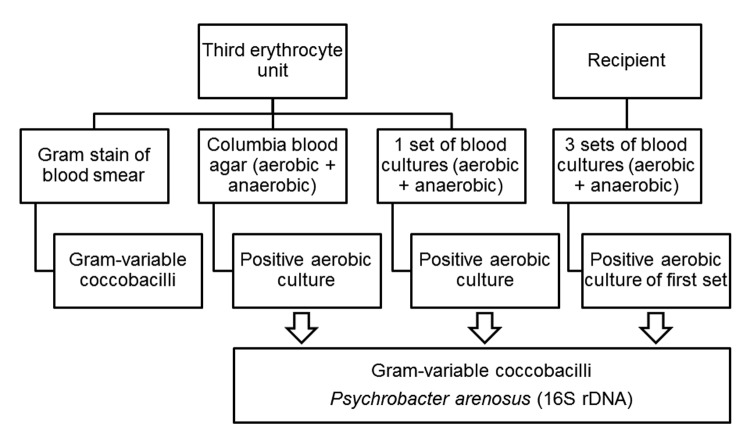
Flow diagram showing samples collected from the blood donor unit (third erythrocyte unit) and a 58-year-old man (transfusion recipient) and results for isolation and identification of *Psychrobacter arenosus*, France.

Molecular identification was performed by 16S rRNA gene amplification and sequencing with fD1 and rP2 primers (*4*), and DNA sequence analysis was performed by using BLAST (www.ncbi.nlm.nih.gov) and leBIBI (http://umr5558-sud-str1.univ-lyon1.fr/lebibi/lebibi.cgi) software. DNA sequences obtained were identical (Genbank accession no. JX416703) and showed 99.7% homology with the *P. arenosus* 16SrDNA sequence previously reported by Romanenko et al. (*5*) (Genbank accession no. AJ609273). Consistent with this identification, subcultures of the isolated strain obtained on tryptic soy agar plates incubated at 4°C, 25°C, and 37°C showed opaque, circular, convex, cream-colored colonies; no subcultures were obtained on Drigalski medium. Phenotypic characteristics of this strain and the strain isolated by Romanenko at al. (*5*) are summarized in the Table. To determine the source of the *P. arenosus* contamination, environmental samples were collected at sites in which erythrocyte units were prepared and stored, but culture results were negative.

Antimicrobial drug susceptibility was determined by using an agar disk diffusion method, and results were interpreted by using MIC breakpoints recommended for other oxidative gram-negative bacilli by the Comité de l’Antibiogramme de la Société Française de Microbiologie (Paris, France) (*6*). The isolate was resistant to lincomycin and susceptible to amoxicillin, amoxicillin/clavulanate, ticarcillin/clavulanate, piperacillin, piperacillin/tazobactam, cefalotin, cefotaxime, ceftazidime, cefpirome, cefepime, imipenem, gentamicin, tobramycin, netilmicin, amikacin, erythromycin, pristinamycin, polymyxin B, trimethoprim/sulfamethoxazole, nalidixic acid, ofloxacin, ciprofloxacin, and fosfomycin.

The patient initially received intravenous ticarcillin/clavulanate (5 g/200 mg, 3×/d) and vancomycin (1g, 2×/d). When the antibiogram was available, treatment was switched to oral administration of amoxicillin/clavulanate (1 g/125 mg, 3×/d) and ofloxacin (200 mg, twice a day) for 12 days, which resulted in rapid recovery.

## Conclusions

*Psychrobacter* species are nonmotile, nonpigmented, aerobic, gram-negative coccobacilli, although Gram staining results are often variable (*7*). These bacteria are psychrotolerant and halotolerant environmental microorganisms (*7*). They have been isolated from many sources, including sea water, ornithogenic soil, air contaminants, fish, poultry, milk, cheese, and irradiated food (*7*). *P. arenosus* was isolated in 2004 from coastal sea ice and sediments in the Sea of Japan (*5*).

*Psychrobacter* species are considered rare opportunistic human pathogens (*8*) and have been isolated from specimens obtained from human blood, cerebrospinal fluid, brain tissue, urine, ears, eyes, vulvae, wounds, and other cutaneous sources (*8*,*9*). *P. phenylpyruvicus* (formerly *Moraxella phenylpyruvica*) has been associated with bacteremia, endocarditis, septic arthritis, foot abscess, and surgical wound infection (*9**–**11**)*. *P. immobilis* has caused fatal infections in a patient who had AIDS (*12*), nosocomial ocular infection (*13*), and meningitidis in a 2-day-old infant (*14*). However, recently the taxonomy of *Psychrobacter* species has been revised, and most human isolates other than *P. phenylpyruvicus* belong to the newly characterized species *P. faecalis* and *P. pulmonis* (*8*). Also, a novel species, *P. sanguinis,* has been isolated from human blood samples (*15*). Thus, the spectrum of human infections associated with the different species of the genus *Psychrobacter* could change rapidly.

We report a case of human moderate septic transfusion reaction caused by *P. arenosus*. The clinical and laboratory findings did not support an acute hemolytic transfusion reaction. Gram staining of a direct smear prepared from the erythrocyte unit showed a high bacterial inoculum, strongly suggesting multiplication of bacteria in this unit before transfusion. *P. arenosus* was isolated from a contaminated erythrocyte unit and blood of the patient obtained after the transfusion was stopped. The patient recovered rapidly after receiving appropriate antimicrobial drug therapy. These findings confirm that the transfusion reaction was attributable to *P. arenosus* contamination of the erythrocyte unit. However, the isolated strain was not identified until 16S rRNA gene amplification and sequencing were performed.

We found differences in biochemical characteristics between this *P. arenosus* strain and the strain isolated by Romanenko et al. (*5*) (Table). *P. arenosus* is able to grow at 4°C–37°C (*5*) and thus could multiply in the erythrocyte unit stored at 4°C for 1 month before transfusion. As in most cases of transfusion-transmitted bacterial infections, source of contamination of the erythrocyte unit was not identified. *P. arenosus* could not be detected in environmental samples collected at sites in which the erythrocyte unit was prepared and stored. As for other gram-negative bacteria, transient bacteremia in an asymptomatic blood donor could be the source of the erythrocyte unit contamination (*1*,*3*), but exogenous contamination at the time of blood collection or preparation of units occurs more frequently (*3*).

**Table:** Characteristics of *Psychrobacter arenosus* isolated in this study (France) and a strain isolated in Russia*

Characteristic	Isolate from this study	Isolate from Russia†
Growth at 5°C	+	+
Growth at 22°C	+	+
Growth at 37°C	+	+
Nitrate reduction	–	–
Urease	–	–
Arginine dihydrolase	–	–
β-galactosidase	–	–
Esculin hydrolysis	–	–
Gelatinase	–	–
Indole production	–	–
Metabolic assay result		
l-arabinose	–	+
Malate	+	+
Citrate	+	+
Caprate	–	–
Acetate	+	+
Propionate	+	+
3-hydroxybutyrate	+	–
Lactate	+	–
Itaconic acid	+	–
l-proline	+	–
l-alanine	+	–
l histidine	+	–
l-serine	+	UNK
Valeric acid	+	UNK
Adipic acid	–	UNK
3-hydroxybenzoate	–	UNK
4-hydroxybenzoate	–	UNK
l-fucose	–	UNK
Gluconate	–	UNK
2-ketogluconate	–	UNK
n-acetylglucosamine	–	UNK
d-glucose	–	UNK
Glycogen	–	UNK
Inositol	–	UNK
Malonate	–	UNK
d-maltose	–	UNK
d-mannitol	–	UNK
d-melibiose	–	UNK
d-mannose	–	UNK
Phenylacetate	–	UNK
l-rhamnose	–	UNK
d-ribose	–	UNK
d-saccharose	–	UNK
Salicin	–	UNK
d-sorbitol	–	UNK
Suberic acid	–	UNK

*+, positive; –, negative; UNK, unknown. †Romanenko et al. (*5*).

*Psychrobacter* spp. strains are highly susceptible to antimicrobial drugs; only 1 strain of *P. phenylpyruvicus* was reported to be resistant to penicillin and aztreonam, 2 strains of *P. immobilis* resistant to penicillin (*10*,*13*,*14*), and 1 strain of *P. immobilis* resistant to gentamicin, tobramycin, ampicillin, and lincomycin (*12*). Most human infections have been treated with a third-generation cephalosporin, leading to rapid recovery (*10*,*11*,*14*). One patient who had AIDS died from septic shock, despite appropriate treatment (*12*).

In conclusion, *P. arenosus* should be considered a psychrotolerant bacterial species responsible for transfusion-transmitted bacterial infections, similar to *Yersinia enterocolitica*, *Listeria monocytogenes*, and psychrophilic *Pseudomonas* spp. (*1**,**2*). However, phenotypic identification of *P. arenosus* is problematic and might require amplification and sequencing of its 16S rRNA gene.
